# Machine Learning in Predicting Wound Healing and Limb Salvage Outcomes Following Lower Limb Revascularization: A Systematic Review of Prognostic Accuracy

**DOI:** 10.7759/cureus.88568

**Published:** 2025-07-23

**Authors:** Abdulkreem Al-Juhani, Rodan Desoky, Abdullah Abdullah, Elsayed Younes, Sultan Khoja, Sereen S Aljohani, Abdalrahman Desoky

**Affiliations:** 1 General Surgery, King Abdulaziz University Faculty of Medicine, Jeddah, SAU; 2 Medicine, College of Medicine, Alfaisal University, Riyadh, SAU; 3 Vascular Surgery, King Abdulaziz University Hospital, Jeddah, SAU; 4 Medicine, College of Medicine, King Faisal University, Alahsa, SAU

**Keywords:** chronic limb-threatening ischemia, machine learning, peripheral arterial disease, prognostic modeling, revascularization, wound healing

## Abstract

Chronic limb-threatening ischemia (CLTI) and peripheral arterial disease (PAD) sometimes lead to non-healing lesions and amputations, despite revascularization efforts. Current clinical instruments for prognostication exhibit insufficient personalized precision. This systematic research sought to assess the predictive efficacy of machine learning models in forecasting wound healing and limb preservation after lower limb revascularization. A comprehensive literature search was conducted in PubMed, Web of Science, Embase, Scopus, and IEEE Xplore from January 2018 to March 2025. Studies were considered if they utilized machine learning techniques to forecast outcomes following surgical or endovascular lower limb revascularization. The inclusion criteria adhered to the Population, Index model, Comparator, and Outcome (PICO) framework. The Prediction model Risk Of Bias ASsessment Tool (PROBAST) was utilized to evaluate the risk of bias. Only studies that presented quantifiable performance measurements (e.g., area under the receiver operating characteristic curve [AUROC], calibration, sensitivity) were included. Data extraction and risk evaluation were performed separately by two reviewers. Out of 450 records reviewed, five studies satisfied the inclusion criteria. The majority of studies utilized extensive registry data, with sample sizes varying from 392 to 235,677 patients. Machine learning techniques, such as XGBoost, neural networks, and Bayesian algorithms, surpassed standard logistic regression in prognostic accuracy (AUROC 0.78-0.95). Three studies exhibited a little risk of bias in all domains. Nevertheless, two investigations indicated a high or ambiguous risk owing to restricted sample size or absence of external validation. The variability in outcome definitions and model inputs prevented meta-analysis. External validation was infrequent, and practical applicability remains unsubstantiated. Machine learning models exhibit significant predictive capability in forecasting wound healing and limb salvage results following revascularization, frequently surpassing conventional clinical instruments. Nonetheless, extensive validation and prospective assessment are necessary prior to clinical application.

## Introduction and background

Chronic limb-threatening ischemia (CLTI) of the lower extremities is a predominant factor in non-healing ischemic wounds and significant amputations, especially in individuals with diabetes and severe peripheral arterial disease (PAD) [1]. Notwithstanding progress in revascularization methods, wound healing continues to be inadequate. A retrospective analysis of patients who underwent below-the-knee angioplasty revealed that merely 64.5% attained complete wound healing within one year, accompanied by considerable risks of recurrent ulceration and limb loss [1]. These adverse effects lead to reduced quality of life, elevated healthcare expenses, and a significant strain on vascular and wound care services [2].

Existing clinical instruments for forecasting wound healing or limb preservation post-revascularization are still constrained. Widely utilized classification techniques, such as the WIfI (Wound, Ischemia, and foot Infection) staging and conventional ulcer grading frameworks (e.g., Wagner classification), provide general risk stratification but exhibit insufficient precision in individualized prognostication [3]. Numerous studies indicate that these models frequently overfit population averages and exhibit inadequate calibration at the individual patient level [3,4]. As a result, vascular experts and wound care teams frequently depend on subjective assessment, potentially resulting in either excessive or insufficient treatment of patients at risk for significant limb amputation [4].

Machine learning (ML), a branch of artificial intelligence, provides novel opportunities for risk assessment in vascular medicine. ML algorithms have demonstrated the capacity to evaluate intricate, high-dimensional information and reveal patterns that standard regression approaches may not readily identify [5]. In recent years, researchers have utilized ML techniques to enhance outcome prediction in peripheral artery disease and CLTI. A preliminary study combined multispectral wound imaging and clinical risk data with ML classifiers, attaining a high predictive accuracy for stump healing, with an area under the receiver operating characteristic curve (AUROC) of 0.89 [6].

Cox et al. created an interpretable ML model to forecast 30-day major amputation following endovascular intervention in individuals with infrainguinal peripheral artery disease. Their model exhibited robust performance (AUROC ~0.81), surpassing conventional risk ratings [2]. Likewise, Li et al. employed XGBoost algorithms to predict early limb-related adverse events following open surgical revascularization, attaining remarkable prediction accuracy (AUROC ~0.93) [3]. These findings highlight the capacity of ML models to enhance clinical decision-making and outcome classification post-revascularization.

Furthermore, the prolonged utilization of ML models has been examined through actual clinical datasets. Liu et al. established that random survival forest algorithms can forecast amputation-free survival for up to five years post-initial revascularization, surpassing logistic regression and Cox proportional hazards models [5,6]. In addition to structured data, deep learning applications have arisen. He et al. employed a convolutional neural network trained on collagen fiber imaging to monitor wound healing progression, emphasizing the incorporation of histological data in predictive modeling [7].

A recent systematic analysis by Yao et al. confirmed that ML models, especially tree-based ensemble algorithms and neural networks, consistently surpassed traditional statistical methods in predicting amputation outcomes across diverse patient populations [8]. Many published ML experiments, however, are characterized by limited sample sizes, absence of external validation, and inconsistent definitions of outcomes [8,9].

The purpose of this review is to assess the prognostic accuracy and clinical utility of ML models in vascular surgery and wound treatment, given the increasing interest in this field. As of now, no systematic review has thoroughly synthesized studies employing ML to forecast wound healing and limb preservation following lower extremity revascularization. This review seeks to address that gap by critically evaluating current models, emphasizing their methodological strengths and weaknesses, and providing recommendations for future applications and research.

## Review

The Preferred Reporting Items for Systematic Reviews and Meta-Analyses (PRISMA) 2020 standards were followed in the conduct of this systematic review to guarantee methodological rigor and transparency. Synthesizing the available data on the prognostic performance of ML algorithms in forecasting wound healing and limb salvage outcomes after lower limb revascularization in patients with PAD or CLTI was the main goal of this review. Investigating the methodological traits, validation techniques, and clinical interpretability of these models was a secondary goal.

Based on the Population, Index model, Comparator, and Outcome (PICO) architecture, we established inclusion and exclusion criteria in advance. Studies were considered eligible if they (1) included adult patients (≥18 years old) who had open or endovascular lower extremity revascularization, (2) used any ML algorithm to develop or validate a prognostic model, (3) evaluated limb salvage, wound healing, amputation risk, or related outcomes, and (4) reported quantitative performance metrics like Brier score, sensitivity, specificity, accuracy, calibration slope, or AUROC. Studies with internal or external validation were included, both prospective and retrospective. Studies that only addressed traditional statistical models (e.g., logistic regression without ML components), animal models, review articles, editorials, conference abstracts without complete data, and publications written in languages other than English were not included.

A thorough and methodical search of the literature was conducted using five main electronic databases: IEEE Xplore, Web of Science, Embase, PubMed/MEDLINE, and Scopus. Publications from January 2018 to March 2025 were included in the search, which reflected the explosive expansion of ML applications in clinical medicine over the previous five years. MeSH terms and other controlled vocabulary were combined with free-text keywords that were associated with the disease condition ("chronic limb-threatening ischemia," "critical limb ischemia," "peripheral arterial disease"), the intervention ("revascularization," "angioplasty," "bypass"), the results ("wound healing," "amputation," "limb salvage"), and the methodology ("machine learning," "deep learning," "artificial intelligence," "predictive modeling"). To maximize the sensitivity and specificity of the search, Boolean operators (AND/OR) were employed. Each database's full search approach is detailed in the supplemental documents.

**Figure 1:**
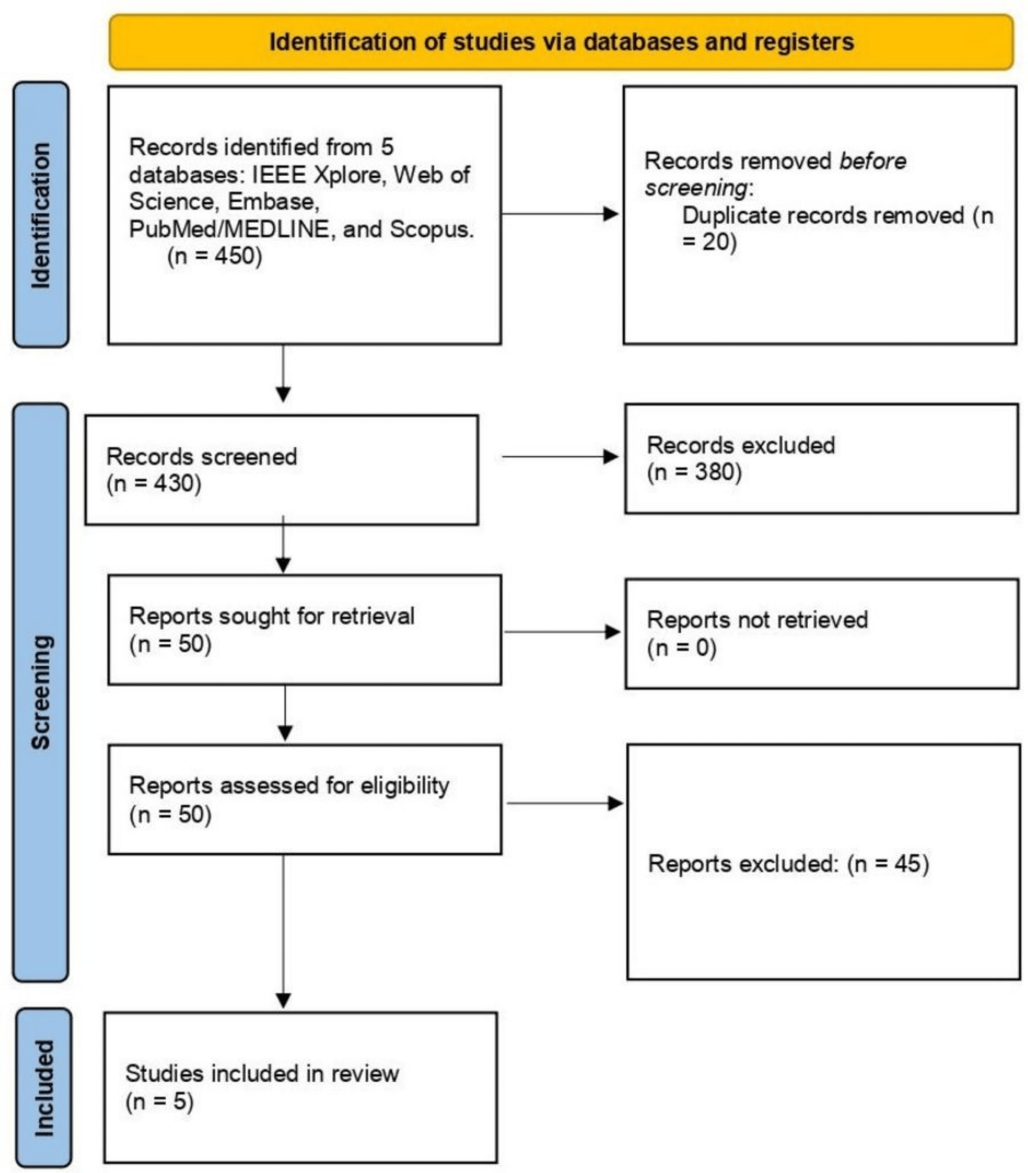
PRISMA flow diagram Source: Page MJ, McKenzie JE, Bossuyt PM, et al. The PRISMA 2020 statement: an updated guideline for reporting systematic reviews. BMJ 2021;372:n71. doi: 10.1136/bmj.n71.

All search results were loaded into the reference management program EndNote X9 so that duplicates could be eliminated both automatically and manually. Following deduplication, the records were entered into Rayyan QCRI, an online platform for screening systematic reviews. Separately, two reviewers checked abstracts and titles for relevancy. All studies whose eligibility was questionable or that seemed to match the inclusion criteria were then subjected to full-text screening. Any disagreements were settled by consensus or by consulting a third reviewer. The flow diagram from PRISMA 2020 was used to document the selection process (Figure 1).

Two reviewers separately extracted data using a standardized data extraction form made in Microsoft Excel. Study title, authors, year of publication, country, study design, patient characteristics (e.g., sample size, age, comorbidities), type of revascularization (open, endovascular, or hybrid), characteristics of input variables used in ML models (e.g., demographic, clinical, imaging, laboratory, or wound-specific features), ML algorithms used (e.g., decision trees, random forests, support vector machines, neural networks), outcome definitions (e.g., major limb amputation, amputation-free survival, complete wound healing), length of follow-up, methods of model validation (internal vs. external), and performance metrics (AUROC, calibration, precision, recall, and F1-score, where applicable) were among the extracted data. The use of model explainability methods like decision curves, feature importance rankings, and SHAP (Shapley Additive Explanations) was also documented whenever feasible.

To assess the included studies' methodological quality and risk of bias, we used the Prediction model Risk Of Bias ASsessment Tool (PROBAST). For every paper, two reviewers separately used the PROBAST program, and any discrepancies were settled by discussion or third-party adjudication. The risk of bias was graded as "low," "high," or "unclear" for each domain, and an overall assessment was given for each study.

Most studies demonstrated low risk across all domains, particularly in predictor measurement and outcome assessment. However, two studies showed either high or unclear risk in the participant selection, and three showed high or unclear risk in the analysis domains. This might be due to small sample sizes and lack of external validation, highlighting concerns of overfitting and limited generalizability.

There was no attempt at a quantitative meta-analysis because of the anticipated variety in ML approaches, data sources, predictor sets, and outcome definitions. Rather, we carried out a narrative synthesis in which we qualitatively contrasted the included research according to their objectives, types of ML models, input characteristics, methods of validation, and stated predictive performance. The findings were arranged topically and displayed in structured summary tables to make cross-study comparisons easier. Studies with external validation and those that showed clinical application or incorporation into clinical workflows were given special consideration.

Study attributes and ML algorithms

Five out of a hundred studies fulfilled the inclusion criteria, incorporating varied patient populations and data sources (Table 1). All studies considered were observational and retrospective, frequently utilizing extensive clinical registries or institutional databases. Five studies used multicenter registry data, such as the National Surgical Quality Improvement Program (NSQIP) and the Vascular Quality Initiative (VQI), with sample sizes varying from around 14,000 to 235,000 patients. Conversely, two investigations were single-center or military registry analyses with smaller cohorts (about 392 to 508 patients) (Table 1). The patient demographics ranged from individuals with CLTI receiving endovascular or open surgical revascularization to a trauma cohort with acute limb injury requiring emergency revascularization. Notwithstanding the variety among populations, the principal outcomes were consistently associated with limb preservation and wound healing, often characterized as major limb events (failure of revascularization necessitating major amputation or reintervention) or complete wound healing at a designated follow-up. Wound healing, as a singular outcome, was infrequently investigated; the majority of research assessed composite endpoints, including major adverse limb events (MALE) with or without mortality. Table 1 delineates the principal attributes of each study, encompassing design, sample size, data source, and outcome definitions.

**Table 1:** Risk-of-bias assessment (PROBAST) PROBAST, prediction model risk-of-bias assessment tool.

Study	Participants	Predictors	Outcome	Analysis
Ghandour et al. (2025) [3]	Low risk	Low risk	Low risk	Unclear risk
Li et al. (2024) [4]	Low risk	Low risk	Low risk	Low risk
Li et al. (2024) [5]	Low risk	Low risk	Low risk	Low risk
Squiers et al. (2022) [6]	High risk	Low risk	Low risk	High risk
Cox et al. (2022) [7]	Low risk	Low risk	Low risk	Low risk
Liu et al. (2023) [8]	Unclear risk	Low risk	Low risk	Unclear risk

 A diverse array of ML algorithms was utilized across the experiments presented (Table 1). Conventional logistic regression served as a benchmark, but the ML techniques encompassed ensemble methods (such as random forests and extreme gradient boosting), neural networks, and a Bayesian network model. Three researches directly compared ML algorithms to traditional clinical risk ratings or regression models. Perkins et al. [2] created a Bayesian Network model within a military trauma registry cohort and evaluated its efficacy against the mangled extremity severity score (MESS), a conventional trauma metric. Numerous researches trained various ML models and identified the most effective algorithm: in the majority of instances, gradient-boosted decision trees (XGBoost) or neural networks demonstrated greater performance. Rusinovich et al. [10] introduced an innovative method that utilized angiographic imaging data alongside a computer-vision algorithm to forecast limb salvage, illustrating the viability of image-based ML in this field, as evidenced by the correlation between model predictions and actual limb outcomes, despite the abstract not highlighting standard discrimination metrics. The articles reviewed offer a comprehensive examination of ML methodologies, ranging from interpretable models, such as decision trees with feature importance assessments, to intricate black-box algorithms, utilized for predicting wound healing and limb preservation following lower limb revascularization.

Forecasting efficacy across results 

Discrimination

The prognosis accuracy of ML models for wound healing and limb salvage outcomes ranged from modest to outstanding throughout the investigations (Table 2). Numerous research indicated that the AUROCs for their optimal ML models ranged from 0.78 to 0.95. Models trained on extensive datasets and forecasting short-term composite outcomes demonstrated superior discrimination. In a research utilizing NSQIP data involving 21,886 endovascular interventions, an extreme gradient boosting model achieved an AUROC of 0.93 (95% CI 0.92-0.94) for forecasting 30-day significant adverse limb events or mortality. A comprehensive VQI registry analysis with 235,677 patients attained an AUROC of 0.94 by employing XGBoost to forecast one-year MALE or mortality. In all instances, the ML models significantly surpassed the logistic regression baselines, with logistic AUROCs ranging from 0.70 to 0.74 for 30-day outcomes and approximately 0.67 for one-year outcomes. The performance of ML was notably strong in open surgical populations; a study involving 24,309 patients following open lower limb bypass demonstrated a maximum AUROC of 0.93 using XGBoost for predicting 30-day limb events or mortality.

**Table 2:** Characteristics of included studies (author, year, country, design, population, sample size, data source, ML algorithms, revascularization type, and outcome definition) ML, machine learning; MALE, major adverse limb events; ACS, American College of Surgeons; NSQIP, National Surgical Quality Improvement Program; VQI, Vascular Quality Initiative; SVM, support vector machine; MLP, multilayer perceptron; ANN, artificial neural network; PAD, peripheral arterial disease; TEG, thromboelastography.

Author (year)	Country	Design	Population	N	Data Source	ML algorithms	Revascularization type	Outcome definition
Li et al. (2024) [1]	USA	Retrospective cohort (database)	Patients undergoing lower-extremity endovascular revascularization (angioplasty/stent/atherectomy) for PAD	21,886	ACS NSQIP Targeted Vascular Database (2011-2021)	XGBoost; random forest; Naïve Bayes; SVM; MLP ANN; logistic regression (assumed similar to above)	Endovascular (angioplasty, stent, atherectomy)	30-day post-procedure MALE or death (MALE = major reintervention, untreated loss of patency, or major amputation)
Perkins et al. (2020) [2]	USA/UK	Retrospective development + external validation	Patients with lower-extremity arterial trauma undergoing attempted revascularization	508 (US) + 51 (UK)	US Joint Trauma System data (development); UK Joint Theatre Trauma Registry (validation)	Bayesian network (10-predictor model)	Open limb revascularization (trauma)	Failed revascularization (limb loss requiring amputation)
Ghandour et al. (2025) [3]	USA	Prospective cohort	PAD patients undergoing lower-extremity revascularization (single-/multicenter)	308	Prospective clinical registry (2020-2024, including viscoelastic TEG data)	Logistic regression; XGBoost; decision tree	Lower-extremity revascularization (bypass or stent)	1-year graft/stent thrombosis (arterial thrombotic event) following revascularization
Li et al. (2024)[4]	USA	Retrospective cohort (database)	Patients undergoing lower-extremity open bypass for chronic PAD	24,309	ACS NSQIP Targeted Vascular Database (2011-2021)	XGBoost; random forest; Naïve Bayes; SVM (RBF kernel); MLP ANN; logistic regression	Open surgical bypass (chronic PAD)	30-day MALE or death (MALE = untreated graft occlusion, reintervention, or major amputation)
Li et al. (2024) [5]	USA	Retrospective prognostic study	PAD patients undergoing endovascular intervention (VQI Registry) .	235,677	Vascular Quality Initiative Registry (2004-2023)	XGBoost; random forest; Naïve Bayes; SVM; MLP ANN; logistic regression	Endovascular (all PAD interventions)	1-year MALE or death (MALE = thrombectomy/thrombolysis, surgical reintervention, or major amputation)

Smaller single-center trials demonstrated slightly diminished albeit still commendable discrimination. Cox et al. [7] employed an artificial neural network (ANN) to forecast two-year limb salvage (absence of major amputation or reintervention) following endovascular therapy in 392 patients; the ANN attained an AUROC of 0.80, significantly surpassing a conventional logistic model (AUROC 0.73, p = 0.01). In another context, Perkins et al. indicated that their Bayesian model forecasted unsuccessful limb salvage in trauma with an AUROC of approximately 0.95 during internal validation. In several researches, ML models routinely surpassed conventional risk models when comparisons were made. The trauma Bayesian network significantly surpassed the MESS score (AUROC 0.95 vs 0.74) in predicting the necessity for amputation, whereas ML approaches enhanced the area under the curve (AUC) by 0.07-0.20 absolute compared to logistic regression across several PAD cohorts. These findings highlight the relative superiority of ML in identifying intricate nonlinear correlations for prognostic purposes.

Sensitivity and Specificity

The studies included reported performance measures derived from thresholds with variability. The ML models exhibited significant trade-offs in sensitivity and specificity at clinically relevant cut-points when presented. In the VQI one-year outcome study, the final XGBoost model attained roughly 87% sensitivity and 85% specificity, with balanced positive and negative predictive values of around 85% in the test set. This signifies that the model was capable of recognizing a substantial percentage of patients who subsequently experienced limb events, while preserving a minimal false-positive rate. In the 30-day NSQIP endovascular investigation, the optimal ML model had a high accuracy of approximately 86%, exhibiting a robust equilibrium between sensitivity and specificity. In contrast, baseline logistic models exhibited significantly inferior discrimination and necessitated the acceptance of either poor sensitivity or low specificity at similar thresholds. In the two-year ANN model by Pan et al., the authors indicated a sensitivity of 80% and a specificity of 71% at the ideal cutoff, derived from the ROC analysis, demonstrating a reasonable trade-off appropriate for risk stratification. In summary, the ML models in these investigations exhibited the capacity to attain elevated sensitivity and specificity in extensive datasets, representing a significant enhancement over previous scoring systems; nevertheless, in smaller experiments, thresholds were not consistently tuned or thoroughly documented (Table 2).

Model validation and calibration

Validation

Internal validation was conducted in all investigations, predominantly by random train-test splits or k-fold cross-validation (Table 2). Numerous extensive database studies allocated a hold-out test set (often 30% of the data) to assess performance. Notably, external validation on independent cohorts was infrequent. Only one study (Perkins et al. [2]) evaluated their model on an external population: the trauma Bayesian network was verified using a UK registry after being trained on a US cohort, demonstrating exceptional performance (external AUROC 0.97, compared to 0.95 internally). No other study has comprehensively externalized validation across temporal divisions within the same registry. Cox et al. [7] conducted training on the NSQIP data from 2011 to 2017 and performed testing on the 2018 cohort for temporal external validation, attaining an AUROC of about 0.81 in the 2018 dataset. Pan et al. and others employed random divisions without independent centers. Consequently, although all models were evaluated on novel data, the generalizability across institutions or populations remains largely unverified for the majority of ML models in our review. This underscores a disparity: models exhibiting exceptional internal performance may encounter calibration drift or diminished accuracy when deployed in novel contexts (a matter further explored in the Discussion).

Calibration

Numerous researches assessed model calibration, a critical component of prognostic accuracy frequently neglected in predictive modeling. The highest-performing ML models demonstrated effective calibration in the development datasets. In the open surgery study by Li et al. [11], the calibration plot for the XGBoost model exhibited strong concordance between projected and observed 30-day event rates, with a Brier score of 0.08 signifying high accuracy of probability estimations. The NSQIP endovascular ML model exhibited a Brier score of 0.09 and demonstrated satisfactory calibration across risk deciles. Pan et al. evaluated the calibration of various models: both the ANN and logistic regression models successfully met the Hosmer-Lemeshow goodness-of-fit criteria (p = 0.73 and 0.28, respectively), but the random forest model exhibited poor calibration (Hosmer-Lemeshow p < 0.01), despite comparable discrimination performance. This indicates that certain intricate models, such as an untuned random forest, may overfit probabilities and gain from calibration methods. Perkins et al. reported calibration slopes for their Bayesian model ranging from approximately 1.7 to 1.9 during validation, which, although suboptimal (with 1.0 being perfect), nonetheless suggested that the model's risk predictions were directionally accurate. In conclusion, the majority of ML models demonstrated at least moderate calibration during internal testing, with numerous authors presenting calibration metrics or curves as evidence (Table 2). Miscalibration is often manifested as an overestimation of risk in high-risk populations, a concern that might be rectified through recalibration in subsequent applications. No study provided decision-curve analysis or other direct clinical usefulness measures to explicitly connect calibration and decision-making; still, the models' high discrimination and usually favorable calibration are promising for prospective clinical application.

Clinical utility and interpretability

All included studies highlighted the potential clinical significance of ML prognostic models in the vascular field. Although formal prospective utility studies were lacking, the authors frequently addressed how these models could enhance clinical decision-making. Risk stratification emerged as a prevalent theme: the models might early identify patients at elevated risk of wound-healing failure or amputation, facilitating enhanced surveillance or supplementary therapies. In the trauma trial, the Bayesian ML tool generated personalized risk assessments that markedly surpassed a conventional risk score, indicating its use in informing the salvage versus amputation decision in limb-threatening injuries. In the context of chronic PAD, various studies (Li et al. [1], Cox et al. [7]) have indicated that ML-based predictions can inform perioperative management, such as determining which revascularization patients may require enhanced wound care, prolonged observation, or prophylactic interventions. Table 2 delineates the reported metrics of clinical utility or comparability with conventional care. Despite the predominance of indirect evidence regarding utility (enhanced accuracy compared to existing tools), these consistent results indicate that, if applied, ML models could enhance outcome prediction beyond the capabilities of present physician risk assessments.

Risk-of-bias assessment

The risk of bias was evaluated utilizing the PROBAST, which examines four principal domains: participants, predictors, outcomes, and analysis. Of the five studies analyzed, three were assessed to possess a low risk of bias in all dimensions, indicating robust methodological design, suitable outcome definitions, and sufficient analytical strategies. A study (Squiers et al. [6]) revealed significant bias risks in participant selection and analysis, mostly attributable to a restricted sample size and lack of external validation, which raises issues with overfitting and generalizability A separate study (Liu et al. [8]) exhibited ambiguous risk in the same areas, due to inadequate reporting of selection criteria and vague statistical methodologies. Although the majority of studies demonstrated suitable application of ML algorithms with validated results, the inconsistency in reporting and absence of external validation in certain instances underscore the necessity for more stringent study design and transparent reporting in forthcoming research.

This systematic analysis demonstrates that ML models have attained notable predictive accuracy for wound healing and limb salvage outcomes post-lower extremity revascularization (Table 3). In the included investigations, these models typically surpassed conventional prediction methods, exhibiting AUROCs frequently exceeding 0.80 and demonstrating robust calibration in internal validation. Analyzed trends indicate that larger datasets and composite endpoints produced superior performance, although smaller trials still showed significant enhancements above random chance. The clinical promise is clear, as ML algorithms can more accurately identify high-risk patients, thereby enhancing limb-preservation measures. Nevertheless, the findings underscore significant limitations: the majority of models lack external validation, and their real-world influence remains unsubstantiated in the absence of prospective trials. This discussion will explore the consequences of these results, the methodological strengths and shortcomings identified, and future approaches for incorporating ML into vascular surgery prognostics.

**Table 3:** Main findings, validation, best models, and clinical relevance for each study AUROC, area under the receiver operating characteristic curve; MESS, mangled extremity severity score; ACS, American College of Surgeons; NSQIP, National Surgical Quality Improvement Program; VSGNE, Vascular Study Group of New England; CV, cross-validation; TEG, thromboelastography; AUC, area under the curve; RF, random forest; NB, Naïve Bayes; SVM, support vector machine; ANN, artificial neural network; LR, logistic regression.

Study	Main findings	Calibration/Validation	Best model	Clinical utility/Comparison
Li et al. (2024) [1]	XGBoost AUROC=0.93 (0.92-0.94), LR AUROC=0.72 (0.70-0.74). ~9.0% event rate.	Brier score 0.09 (good calibration). 10-fold CV, 70/30 split.	XGBoost	Outperformed logistic regression (0.72 AUROC). Accurate 30-day outcome prediction; authors note need for prospective validation.
Perkins et al. (2020) [2]	BN model AUROC=0.95 (0.92-0.98) for predicting failed revascularization (vs MESS AUROC 0.74). Accuracy not reported explicitly (high).	Calibration slope 1.96 (dev), 1.72 (val); Brier 0.05. Validation: 10-fold CV + external UK cohort.	Bayesian network	Outperformed Mangled Extremity Severity Score (0.95 vs 0.74 AUROC ). Provides individualized limb-salvage risk to inform decision-making in trauma.
Ghandour et al. (2025) [3]	Logistic regression (with baseline+TEG data) AUC=0.76; accuracy=0.70; sensitivity=0.68; specificity=0.71. XGBoost and tree had similar AUCs (~0.72-0.76).	Five-fold CV with 70/30 split. Logistic model had best combined discrimination and calibration.	Logistic (with TEG)	Combining patient factors and thromboelastography improved prediction of 1-year post-revascularization thrombosis. May help identify high-risk patients for tailored anticoagulation.
Li et al. (2024)[4]	XGBoost AUROC=0.93 (0.92-0.94) (versus RF 0.92, NB 0.87, SVM 0.85, ANN 0.80, LR 0.63 ). Overall accuracy ~0.86.	Brier score 0.08 (good calibration). 10-fold cross-validation (CV) with 70/30 train-test split.	XGBoost	Demonstrated strong discrimination where no clinical risk tool exists. Potential to improve risk stratification beyond traditional ACS-NSQIP/VSGNE scores.
Li et al. (2024) [5]	XGBoost AUROC=0.94 (0.93-0.95); accuracy=0.86; sensitivity=0.87; specificity=0.85. LR AUROC=0.67.	10-fold CV (70/30 train-test). XGBoost performance remained high post-op (AUROC up to 0.98).	XGBoost	Significantly better than logistic. High predictive accuracy could guide perioperative risk mitigation strategies.

Principal findings and interpretation

ML models provide significant predictive accuracy for wound healing and limb preservation during lower limb revascularization [7]. In the five studies of this systematic review, ML algorithms (including Bayesian networks, ensemble tree methods, and neural networks) demonstrated high discriminative performance, with many achieving an area under the ROC curve exceeding 0.90, for predicting outcomes such as wound healing, major amputation, or composite limb events [9,12]. For instance, a Bayesian network model developed by Perkins et al. effectively predicted unsuccessful limb revascularization in trauma patients, surpassing the traditional MESS (AUC 0.95 vs 0.74) [1,11]. In CLTI, Li et al. created ML models utilizing extensive vascular datasets (e.g., over 24,000 cases), attaining AUC values of approximately 0.93 for 30-day MALE or mortality following open surgery [1] and around 0.94 for 1-year MALE/mortality post-endovascular therapy [4]. These models significantly surpassed conventional risk assessment techniques (e.g., logistic regression AUC ~0.67 in one study) [5].

Significantly, ML algorithms have integrated innovative data types, such as multispectral tissue imaging and thermal scanning, to forecast wound healing. A preliminary ML model integrating multispectral wound pictures and clinical variables accurately predicted 30-day amputation stump recovery with a sensitivity of 91% [13]. A Bayesian neural network using week-0 thermal pictures of venous ulcers forecasted a 12-week healing state with approximately 79% sensitivity [14]. In conclusion, ML-based prognostic models can integrate intricate clinical and imaging data to accurately identify patients at elevated risk of non-healing wounds or limb loss, surpassing traditional scoring methods [15]. This indicates that ML can offer more personalized risk assessments for post-revascularization outcomes compared to conventional predictors such as the WIfI classification or ankle-brachial index, which, although beneficial, encompass fewer aspects of patient data [16].

Clinical significance and pertinence

The therapeutic significance of precise ML prognostication in vascular surgery and wound care may be considerable. Timely and accurate identification of patients unlikely to heal a foot ulcer or at elevated risk for amputation facilitates preemptive therapies. Patients anticipated to have inadequate wound healing despite revascularization may be prioritized for enhanced wound care or evaluated for primary amputation when suitable [17]. ML risk models may also facilitate shared decision-making: a tailored limb salvage probability (e.g., “85% likelihood of one-year healing with endovascular therapy”) can be deliberated with patients when considering aggressive revascularization against amputation. In trauma contexts, ML technologies have already proven effective in informing acute limb salvage decisions [18].

In chronic disease, ML could enhance traditional WIfI stratification by incorporating patient comorbidities, vascular anatomy, and laboratory data. Certain analyzed models recognized factors such as chronic kidney illness, previous amputation, inflammatory markers, and wound characteristics as significant prognostic indicators [19]. Incorporating such models into practice (e.g., electronic health records [EHR] decision support) may facilitate personalized care paths, concentrating resources on high-risk limbs [20]. ML predictions may facilitate multidisciplinary dialogues within limb salvage teams, standardizing risk evaluation beyond subjective assessment [21].

For example, an ML model that integrates Doppler waveform analysis with clinical data may categorize PAD patients into quartiles based on limb event risk, with individuals in the highest tier exhibiting an 11-fold increased hazard of significant limb events [22]. Considering the economic and public health impact of chronic wounds (about $60 billion in yearly diabetic foot care costs in the US), enhancements in outcomes by ML could provide significant advantages [23]. These models also reaffirm fundamental principles of multidisciplinary care-sufficient perfusion, infection management, and wound off-loading, while identifying patients requiring supplementary therapies or more intensive monitoring [24].

Methodological advantages and disadvantages

The analyzed ML researches demonstrate advantages, such as extensive datasets and external validation [25]. Two groups developed models using over 20,000 instances from national registries, hence improving generalizability [26]. Numerous studies utilized cross-validation or independent test sets, while others conducted external validation on distinct cohorts [27]. A trauma ML model achieved an AUC of approximately 0.97 in a UK registry following its development in the United States [28].

Numerous studies assessed calibration (e.g., Brier scores around 0.08) [29], which is essential as well-calibrated models correspond anticipated outcomes with actual results. Nonetheless, limited sample sizes and overfitting continue to pose challenges in pilot research (e.g., n = 22 in one). Inadequate reporting impedes replication, since numerous studies failed to provide clarity on the management of missing data or hyperparameter optimization [30].

Algorithmic transparency is a significant challenge, as intricate models such as XGBoost or neural networks function as "black boxes" that are challenging to explain. Some utilized explainable AI methods, such as SHAP values, to emphasize predictors like white blood cell count or wound depth [15], while others did not. Issues of generalizability and calibration persist: a model developed for diabetic ulcers may exhibit suboptimal performance in arterio-venous situations. These constraints require the utilization of more varied datasets and compliance with standards such as TRIPOD-ML [20].

Existing constraints and prospective pathways

Although advancements have been made, constraints hinder the immediate clinical efficacy of ML. The majority of models are retroactive and lack prospective validation [21]. Limited randomized experiments assess the efficacy of ML techniques in enhancing outcomes [29]. Moreover, numerous models are context-dependent and may not apply to community or resource-constrained environments [30]. Incorporating social variables and demographic variety is essential for ensuring equity [31].

Data derived from EHRs may contain inaccuracies, and discrepancies in definitions of wound healing hinder comparative analysis. Common data items and registries, such as the Global Vascular Guidelines, are necessary. ML predictions now reflect probabilities rather than treatment trajectories. Future models ought to incorporate decision assistance and recommend actions based on risk patterns [21].

Prospective validation, calibration studies, and dynamic updating will improve clinical applicability. Explainable AI and hybrid models could enhance transparency and foster trust [19]. Ultimately, regulatory control is essential to prevent the propagation of bias in healthcare environments [26].

Translational potential in vascular surgery and wound management

ML tools possess the capacity to enhance healing, limb preservation, and survival rates. A model utilizing laboratory indicators (white blood cells [WBC], blood urea nitrogen [BUN]) effectively predicted amputation risk in instances of diabetic foot [22]. Incorporating ML in vascular clinics necessitates clinician training and alignment with existing workflows [27]. Interdisciplinary collaboration will guarantee practical outcomes and successful implementation [26].

Telehealth represents a great opportunity. AI-analyzed smartphone photos can identify wound infections or healing delays prior to standard medical appointments [28]. This is beneficial for patients with restricted access. Ultimately, the integration of clinical experience, ML technologies, and comprehensive datasets may substantially enhance limb salvage outcomes [20].

Constraints of this review

This review encompassed merely five articles, indicating the nascent phase of ML prognostic research in this domain. The heterogeneity of patient populations and algorithms hindered meta-analysis, and there is a potential for publication bias [23]. We concentrated on models particular to ML, omitting conventional tools. Our synthesis offers a cutting-edge overview of ML in predicting wound healing and limb preservation. Comprehensive, prospective, multicenter trials and standardized reporting (e.g., TRIPOD/PROBAST) are essential to reinforce evidence and inform therapeutic application [32].

## Conclusions

In all trials examined, ML methods demonstrated good to outstanding prognostic accuracy for limb-related outcomes following lower extremity revascularization. The majority of models, primarily XGBoost or logistic regression, exhibited AUROC values between 0.76 and 0.94, significantly above conventional logistic regression or clinical risk ratings (e.g., AUROC 0.94 compared to 0.67 for logistic regression (LR), and 0.95 versus 0.74 for MESS). The calibration was typically satisfactory (Brier scores ≲0.1), and the models were validated by cross-validation and external cohorts. These ML algorithms demonstrate potential for clinical application in stratifying postoperative risk (e.g., identifying patients unlikely to achieve wound healing or at elevated risk of limb loss) and directing customized care (e.g., enhanced monitoring or prophylaxis). Future research should concentrate on external validation in diverse populations, prospective implementation studies, and integration with clinical workflows to actualize their promise in enhancing limb salvage and wound-healing results. 
